# How great is the negative impact of masking and social distancing and how can we enhance communication skills in the elderly people?

**DOI:** 10.1007/s40520-021-01830-1

**Published:** 2021-03-16

**Authors:** Davide Brotto, Flavia Sorrentino, Anna Agostinelli, Elisa Lovo, Silvia Montino, Patrizia Trevisi, Niccolò Favaretto, Roberto Bovo, Alessandro Martini

**Affiliations:** 1 ENT Unit, Department of Neuroscience DNS, University of Padova, Via Giustiniani 2, 35128 Padova, Italy; 2 Padova University Research Center “I-APPROVE - International Auditory Processing Project in Venice” , “Santi Giovanni e Paolo” Hospital, Venice, Venice, Italy

**Keywords:** COVID-19, Communication, Hearing loss, Face masks, Elderly, Hearing aids

## Abstract

During COVID-19 pandemic, protective measures such as social distancing and face masks posed a challenge in daily communication, in this context the elderly are one of the most at risk categories as widely exposed to hearing loss. This article focuses on how the COVID-19 pandemic affected verbal communication, especially on those people that even in normal conditions present an increased difficulty in speech perception. Special attention has been paid to hearing aids and cochlear implant users, these devices indeed can be affected by a speech intelligibility reduction and could be uncomfortable if used together with face masks. Possible alternatives and solutions will be proposed to reduce the negative impacts of face coverings on communication, to enhance speech intelligibility and to manage wearability of hearing rehabilitation devices.

## Introduction

The current COVID-19 pandemic has forced worldwide mandatory adoption of social distancing measures and face masks to limit the diffusion of the SARS-CoV-2 infection via droplets. These actions are crucial to protect the most vulnerable part of the population currently facing the highest number of victims, the elderly people [1].

Even though the implementation of these precautions should be considered essential, their impact on the communicative skills is dramatic. Moreover, two thirds of patients over 70 years old present hearing loss, making this population at high risk for communicative issues even in normal conditions [2]. Noteworthy, although hearing loss can be treated with hearing aids or cochlear implants, the impact is still severe because many patients rely also on the visual clues to properly understand speech. In addition, only a limited part of old patients use hearing aids or cochlear implants due to the limited access to an audiological evaluation, the underestimated possible benefit and the aesthetic issue.

Facial masks are indeed a barrier that impair speech production and perception, make lip reading impossible and facial expressions not visible: consequently, any additional clue to hearing perception is in the end missing. In addition, face masks make it difficult to read emotions from facial expression [3] and communication seem to be further limited in emotional conditions [4].

The impact of the pandemic in terms of diagnostic delay for multiple pathologies and surgical treatment [5], logistic issues in managing chronic diseases and mental health [6], limitations to individual autonomy and freedom is exacerbated by the difficult communication between individuals.

Even patients using hearing aids or cochlear implants face severe difficulties due to the above-mentioned barriers that might lead to limited use of the devices, which may negatively impact these patients’ health.

Although some limits are unbreakable, others can be bypassed with modern technology.

## Audiological impairment and mental health

Facial masks behave like a low-pass filter, attenuating the medium–high frequencies of the speaker’s voice with a reduction from 3 to nearly 12 dB (depending on the mask type) [7]. More importantly, speech discrimination is deteriorated causing difficulty in listening also in subjects with no hearing impairment and the impact in patients with hearing loss is, therefore, dramatic.

Old age is in itself a risk factor to social isolation [8] that may be detrimental to cognitive function [9] and lead to higher amounts of depression and anxiety symptoms [10].

Hearing loss, strictly associated with aging, increases these risks and negatively impacts the quality of life of these subjects; auditory impairment is associated with social isolation and loneliness [11], cognitive decline, dementia and poor mental health [12].

Negative effects of hearing loss can be minimized by auditory rehabilitation with hearing aids or cochlear implants [13, 14]. In recent years, multiple studies have highlighted the benefits of hearing rehabilitation in preventing or delaying cognitive decline and dementia even in very elderly patients.

The benefit has proved effective and should be recommended even in mild hearing loss [15]. Concerning the profound hearing impairments, age is no longer a contraindication to cochlear implantation and the audiological outcome in elderly people is comparable to those obtained in young adults [16].

COVID-19 pandemic has negatively affected older adults, especially for those with hearing impairment reducing their communicative skills, favoring social isolation and compromising their mental health [17]. For these reasons, healthcare personnel and relatives should not give up on the effort to communicate and use the best available instruments to enhance a positive relation with patients.

## Possible alternatives to reduce the impact/to face masks

Face masks are considered to be essential barriers to reduce the diffusion of droplets while breathing or speaking. The usefulness is non-negligible and it is a cost-effective solution. Nonetheless, in some conditions, the necessity to communicate might force both the clinician and the patient to give up on their use, but alternatives must be considered for safety reasons.

The simplest way to effectively communicate with a hearing-impaired patient using face masks is to write the sentences down or on a computer screen, allowing the patients to read them and reply. Despite the safety of this method, time spent for each patient greatly increases, and it cannot be used for speech rehabilitation and therapy, because no auditory information is provided.

However, in this context, the clinician should remind that other options are available to protect him/herself and the patients. Face masks with clear windows are available in the market and provide the same protection of the classic face masks enabling lip reading.

Although their value as barriers is limited, even transparent shields can be considered when both lip reading and observation of facial expression are required, such as in speech therapy. A double barrier can be achieved in this setting if the speech therapist uses both a transparent wearable shield and another, wider transparent screen on the desk. In these conditions, providing an appropriate and continuous change of air can reduce the high density of droplets in the air.

## The best way to enhance speech intelligibility with face masks

In most situations, it is not possible to avoid the use of face masks; consequently, it may be useful to improve the way we interact especially with the elderly people to avoid some of the limitations imposed by face coverings. Specifically, it is important to use a more sustained tone and a lower pitch of voice, because face masks damage high-frequency transmission in speech [7]. In general, the initial failure to communicate verbally should drive the speaker to alter speech rate and vocal intensity, slowing it down and raising the volume [18].

Another coping strategy is to simplify as much as possible the message to reduce the required cognitive effort. In addition, slowing down the rhythm of the eloquium without “chanting” syllables (i.e. repeating the words dividing them in syllables) and repeating the information several times (while keeping the structure of the sentence unchanged) should be considered useful strategies especially in elderly people.

Unfortunately, face coverings impair communication far beyond the acoustic of speech transmission. As highlighted by a recent survey, wearing face masks impacts content of communication, feeling of interpersonal connection and willingness to engage in conversation [19]. All these factors can be crucial when hearing and cognitive effectiveness are reduced, as it is common in old patients.

For this reason, it is important to have a more accurate communication awareness when addressing old people, especially those with hearing loss.

In those cases, the use of gestures and written language provides visual information that can be a helpful source of guidance to enhance auditory comprehension. Also, the use of eyebrows or upper cheeks mimic movements can be helpful in enhancing the emotional messages connected to the speech [20]. Only a face-to-face talk allows a person to optimize reading facial expression and catching the visual cues and gestures provided by the speaker. This is why it is crucial to face the communication partner, limiting as much as possible confounding factors.

## Hearing aids and cochlear implants: how to manage wearability?

The concomitant use of hearing aids and/or cochlear implant and personal protective equipment can be challenging and uncomfortable especially in the elderly people. Nonetheless, their use should be stimulated, even in the current conditions, since it is easier than it seems.

Modern retro-auricular hearing aids have now reached excellent levels of miniaturization and they can be used without problem when glasses and face masks are also present. In-the-ear hearing aids can be even better in these conditions. For cochlear implants users, single-unit processors are available,they are easier to manage, and there is no conflict with glasses or face masks; even retro-auricular sound-processor can be used, for example, it can be clipped directly on clothes, or using silicone laces, customized coupling, or anchoring systems to the auricle. These strategies can prove particularly useful in patients who need an oxygen mask or helmet. For these patients, it may also be advisable to use the appropriate moisture protections to guarantee the correct functioning of the processor. In addition, the sound-processors can be set to use signal LEDs to show if the device is working correctly, making it easy to check if the patient can hear.

## How to enhance speech with hearing aids and cochlear implants?

One of the greatest challenges for hearing aids and/or cochlear implants users is to discriminate language in noise. The goal is not easy, especially for the elderly people. In addition, FFP3 masks combined with a face shield in noisy environments can cause a speech intelligibility reduction of up to 70% [21].

In general, fitting parameters for both hearing aids and cochlear implants should be set to improve speech intelligibility enhancing the high frequencies (those reduced by face masks). In addition, higher levels of volume and specific microphone directionality can be set to compensate for sound attenuation.

There are also several instruments to reduce the problem of background noises that impairs speech intelligibility such as magnetic induction and wireless systems. As a rule, these systems are based on a transmitter that can be a wearable microphone or a connectable device that transmits the hearing input directly to the hearing aid or the cochlear implant, thus limiting the background noise coming from the surrounding environment. The advantage of these systems are that they limit the negative influence of distance, environmental noise and reverberation with the chance of a direct communication with healthcare staff or relatives that can be even distanced by a glass or a window.

The magnetic induction system is a quite “old” but efficient technology used with telephone coil (T-coil). If the hospital is equipped with this technology, the patient can receive the signal from a microphone or a screen directly to the hearing aid or the cochlear implant, without the addition of any device.

Most recently, more and more hearing aids and cochlear implants are available with an integrated wireless receiver that can be directly connected to a wireless microphone (transmitter). This technology enables a connection to telephones, smartphones, multimedia players, stereos, TVs, PCs and tablets without the need of additional devices. The connection is efficient not only for phone calls and FaceTime (R), but also to take advantage of all the Voice Over features for automatic reading of what is displayed on the screen. This may be useful easily to communicate both with the family and with the healthcare staff, even by phone or to support short-distance communication.

In addition, hearing aids and cochlear implant companies have also developed apps for mobile phones, supported by Bluetooth(R) 4.0 or later. These apps permit changing programs or volume, controlling battery life, getting help finding your sound processor if it gets lost and starting wireless streaming all without having to deal with a separate controller but only using the phone.

Magnetic induction and wireless connections are efficient even if obstacles are present (such as wearable shields, screens or even windows) or when the receiver (hearing aid/cochlear implant) is meters away from the transmitter (microphone, TV, phone, etc.), thus allowing to avoid close contacts.

All the abovementioned solutions are easy to manage even for inexperienced people and can be extremely helpful to communicate in complex environments and to avoid patient isolation from family members and healthcare providers.

## Conclusions

Face masks and social distancing are mandatory in the current pandemic time. Limited hearing input can be extremely harmful with possible psychological and cognitive consequences that should be prevented.

Although enhancing hearing performance is not without economic and care costs, the impact on the elderly people is non negligible, and we should all realize that most issues can be faced with communication-awareness and limited personal effort.

The healthcare providers should not give up on the use of hearing aids and cochlear implants since the psychological support is necessary for the patients and also informed consent is based on the clear communications and understanding of the messages.

Moreover, a correct use of the available technology (i.e. wireless systems) can be useful to promote social distancing, without giving up on an efficient oral communication with high-quality hearing input.

## Data Availability

The data that support the findings of this study are available on request from the corresponding author.
